# A Varian DynaLog file‐based procedure for patient dose‐volume histogram‐based IMRT QA

**DOI:** 10.1120/jacmp.v15i2.4665

**Published:** 2014-03-06

**Authors:** Juan F. Calvo‐Ortega, Tony Teke, Sandra Moragues, Miquel Pozo, Joan Casals

**Affiliations:** ^1^ Departamento de Radioterapia Hospital Quirón Barcelona Spain; ^2^ Medical Physics BC Cancer Agency, Centre for Southern Interior Kelowna BC Canada

**Keywords:** DVH, DynaLog, IMRT, QA

## Abstract

In the present study, we describe a method based on the analysis of the dynamic MLC log files (DynaLog) generated by the controller of a Varian linear accelerator in order to perform patient‐specific IMRT QA. The DynaLog files of a Varian Millennium MLC, recorded during an IMRT treatment, can be processed using a MATLAB‐based code in order to generate the actual fluence for each beam and so recalculate the actual patient dose distribution using the Eclipse treatment planning system. The accuracy of the DynaLog‐based dose reconstruction procedure was assessed by introducing ten intended errors to perturb the fluence of the beams of a reference plan such that ten subsequent erroneous plans were generated. In‐phantom measurements with an ionization chamber (ion chamber) and planar dose measurements using an EPID system were performed to investigate the correlation between the measured dose changes and the expected ones detected by the reconstructed plans for the ten intended erroneous cases. Moreover, the method was applied to 20 cases of clinical plans for different locations (prostate, lung, breast, and head and neck). A dose‐volume histogram (DVH) metric was used to evaluate the impact of the delivery errors in terms of dose to the patient. The ionometric measurements revealed a significant positive correlation $\left(R^{2}=0.9993\right)$ between the variations of the dose induced in the erroneous plans with respect to the reference plan and the corresponding changes indicated by the DynaLog‐based reconstructed plans. The EPID measurements showed that the accuracy of the DynaLog‐based method to reconstruct the beam fluence was comparable with the dosimetric resolution of the portal dosimetry used in this work (3%/3 mm). The DynaLog‐based reconstruction method described in this study is a suitable tool to perform a patient‐specific IMRT QA. This method allows us to perform patient‐specific IMRT QA by evaluating the result based on the DVH metric of the planning CT image (patient DVH‐based IMRT QA).

PACS number: 87.55.Qr

## I. INTRODUCTION

Currently, IMRT has become the conventional treatment modality in many radiotherapy departments. Dosimetric treatment parameters, such as monitor units (MUs) and beam fluences, depend on the individual patient plan and can vary substantially among patients as a function of the modulation, requiring a patient‐specific QA for each treatment plan.

Several methods for patient‐specific QA are recommended in ICRU Report 83^(1)^ in order to verify that the intensity pattern will deliver the desired absorbed dose. After commissioning an IMRT technique in our department, we established a patient‐specific QA including two steps. First, the absolute dose from all beams used to treat the patient is measured with an ion chamber at an appropriate point in a phantom (hybrid plan) and compared with the corresponding computed absolute dose (“monitor unit verification”). Second, planar measurements of the intensity pattern (“fluence verification”) from individual beams are recorded using an electronic portal imaging device (EPID) and compared to predictions given by the treatment planning system (TPS).

However, this patient‐specific QA procedure has several drawbacks. From a logistical point of view, in a very busy radiation oncology department with almost all delivered treatments using IMRT, it is very difficult to find a dedicated time slot on the linear accelerator (linac) to perform the required measurements for every IMRT plan. Kruse^(2)^ has recently shown that perbeam planar measurements are insensitive to important dosimetric inaccuracies of the overall plan. As in our case, patient‐specific IMRT quality assurance typically compares measured and calculated dose distributions in phantom, but not on the patient. The clinical importance of the difference between measured and calculated dose is often difficult to interpret. Zhen et al.^(3)^ suggested the use of patient dose‐volume histogram‐based metrics for IMRT QA to estimate the patient dose impact of TPS or delivery errors in terms of the changes in dose‐volume histogram (DVH) values for the target volume and organs at risk (OARs).

Some authors discuss using logs generated by the multileaf collimator (MLC) controller during an IMRT delivery as a tool for inverse dose verification for IMRT patient‐specific QA.^(4)^ These dynamic logs (“DynaLog files”) may be converted to MLC files for recalculation of the patient dose in order to assess the dosimetric information of the delivery. Li et al.^(5)^ pointed out that the use of DynaLog files can be helpful in assessing the clinical impact of potential delivery errors.

It is possible to assess the MLC accuracy during the delivery by just comparing the dynamic MLC files as TPS‐calculated to the MLC‐reconstructed ones (from DynaLog files), but this kind of comparison is not able to assess the impact of the potential MLC errors of the overall IMRT plan on the patient dosage. To have some patient dose metrics, it is necessary to estimate the dose to the patient, and the DynaLog files offer a way to do it.

The aim of this study is to investigate the performance of a patient DVH‐based method implemented in our department for patient‐specific QA as a way to check whether the patient is actually treated using the delivery instructions produced by the planning process and avoiding measurements for each clinical IMRT plan. It is not an objective of this study to assess the accuracy of the AAA model when IMRT plans are computed on Eclipse TPS. This issue has been already studied by several authors.^6^, ^7^

## II. MATERIALS AND METHODS

### A. Treatment planning

In our department, all plans are calculated using the Varian Eclipse TPS (version 10.0; Varian, Palo Alto, CA) with the analytical anisotropic algorithm (AAA). The data presented in this study were obtained using a Varian Clinac 2100 CD equipped with a Millennium 120 leaf MLC and a PortalVision aS500 imager (Varian, Palo Alto, CA). All IMRT plans consisted of fixed field arrangements using a dynamic delivery technique (sliding window). All measurements described in this work were performed in clinical mode at nominal dose rates of 300 and 600 MU/min. The IMRT plans used in this study were delivered using the Aria record and verify system (version 10; Varian).

### B. MLC DynaLog file processing

The MLC controller of the Varian linac creates two DynaLog files (one for each MLC bank; A and B) for each IMRT field delivered. A detailed analysis of the data contained in the MLC DynaLog files was given by Litzenberg el al.^(8)^ The most relevant information contained in the DynaLog files are the dose index (or fractional MU), the segment number, and the calculated and reported positions of each leaf. These data are acquired every 55 ms and recorded at the end of the treatment. A proper MLC calibration is mandatory to ensure that the recorded leaf positions in the log files agree with the actual ones. DynaLog files were used in this study to generate an MLC file (“dva file”) compatible with the Eclipse TPS. This DynaLog‐to‐dva conversion was performed using a MATLAB‐based (version 7.4.0.287; The MathWorks, Inc., Natick, MA) code developed by Teke et al.^(9)^ All dva files were reconstructed using both recorded leaf positions and fractional MUs of the DynaLog files to take into account MLC position and MU.

The original MLC files of a clinical IMRT plan may be replaced by the corresponding dva files in order to calculate the “delivered” dose to the patient. By keeping the same dose calculation algorithm (AAA in our study), the differences between delivered and planned doses can be seen as indicators of errors in the positions of the dynamic MLC leaves that occurred during the treatment delivery. As “delivered” dose to the patient is calculated using the leaf positions and fractional MU recorded in the DynaLog files, it is very important to remark at this point that a dedicated MLC QA program is necessary in order to assure the correct MLC calibration, as well as the correct performance of the MLC controller in recording the log files. Also, it important to indicate that nothing in the method described above actually verifies the patient position.

### C. Accuracy of the DynaLog‐to‐dva conversion process

A crucial point in this study is to assess the accuracy of the MATLAB‐based code used to generate a dva file from its corresponding pair of DynaLog files. To do this task, the DynaLog files recorded during a treatment session were collected for three types of IMRT delivery (prostate, head and neck, and lung cancer). As the DynaLog files contain information about the planned and recorded positions for each leaf, as well as the delivered fractional dose in each segment, the MATLAB code may be modified such that the generated dva file incorporates only the planned leaf positions and the planned fractional values. The created dva files after this code manipulation were imported into Eclipse to replace the original ones, and a second dose array was calculated. The accuracy of the MATLAB‐based code was assessed by comparing the dose distributions for the original plan and the recomputed one. Dose comparison was done for the target volume and the OARs using the DVH metrics described below. The expected dose difference should be zero if the MATLAB code was well programmed.

### D. Patient dose reconstruction procedure

**Figure 1:**
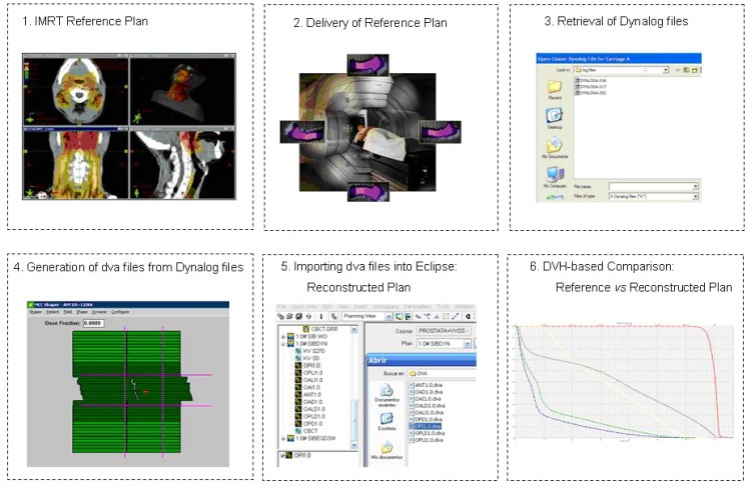
Workflow of the patient dose‐volume histogram‐based IMRT QA procedure.

The proposed procedure is illustrated in Fig 1. After delivery of a clinical plan (“reference” plan), the two DynaLog files (one per bank) for each field are retrieved from the MLC controller and processed to generate the corresponding dva file for the field. Each dva file takes into account the delivered MUs for each field segment and recorded leaf positions according to the information given by the DynaLog files. The resulting dva files are imported into the Eclipse TPS, and the patient dose is recalculated using the AAA algorithm, keeping the monitor units of the reference plan (“DYN” plan). The total number of monitor units delivered by the linac was verified for each field by the Aria record and verify system. In this way, the reconstructed plan could reveal any dosimetric inaccuracy occurring during the dynamic dose delivery. Comparison of the two plans is done via dose‐volume histograms (DVHs) for the target and organs at risk, performing in this way a patient DVH‐based IMRT verification of the reference plan.

The procedure described above, allows the performance of patient‐specific IMRT QA by using the DynaLog files recorded during every treatment session of the patient. This method also offers, at least in the case of fractioned radiotherapy, the possibility of performing patient‐specific IMRT QA without a dedicated time slot to deliver the clinical IMRT plan before the patient treatment. Obviously, in case of a single dose treatment, the DynaLog‐based method should be applied by using a “dry run” session without patient.

At this point, it should be noted that the patient dose distribution obtained as described above does not take into account any patient setup error, unless the patient is perfectly positioned and in the exact position as when scanned for treatment planning. Our patient dose reconstruction procedure is designed to assess the clinical impact of potential errors of the dynamic MLC on the patient dosage.

### E. Validation of the dose reconstruction process

The accuracy of the dose reconstruction method based on DynaLog files was investigated by introducing errors in a reference plan (“plan.ref”) consisting of a typical five‐beam arrangement for treatment of prostate cancer. From the copies of the reference plan, ten intentional errors were introduced (Table 1), such that ten erroneous plans were generated (plan.err1, ….. plan.err10).

The following nine systematic errors were introduced simulating realistic MLC errors:

a) an expansion of the MLC opening with 1.0, 2.0, and 4.0 mm divided equally on each bank;

b) all leaves in bank A were shifted by 0.5, 1.0, and 2.0 mm without modification of the opposite bank in a way that the MLC opening is increased; c) same leaf shifts as described in b), but in bank B.

In addition, an erroneous plan was generated by removing several segments of the MLC file for each field, in order to simulate a potential network failure.

The reference plan and its generated erroneous plans were computed on an acrylic phantom (type 400201; PTW, Freiburg, Germany), previously CT‐scanned and imported into Eclipse. This phantom has several inserts to accommodate an ionization chamber (IC) of 0.125 cm^3^ (31010; PTW), and the active volume of each detector was outlined and the “chamber dose” calculated by the TPS was reported as the mean dose to this volume. After the creation of the plans, two kinds of measurements were performed.

**Table 1:** Comparison of the measured changes vs. the detected ones using the DynaLog‐based method for the isocenter dose. A significant correlation was obtained $\left(R^{2}=0.9993;p<0.01\right)$

*# Error*	*Induced Error*	*Dcalc.DYN/Dcalc.ref*	*Dmeas.error/Dmeas.ref*
1	*1* mm MLC opening	1.038	1.039
2	2 mm MLC opening	1.077	1.077
3	4 mm MLC opening	1.154	1.153
4	0.5 mm shift bank A	1.019	1.019
5	1 mm shift bank A	1.037	1.036
6	2 mm shift bank A	1.074	1.071
7	0.5 mm shift bank B	1.019	1.020
8	1 mm shift bank B	1.039	1.040
9	2 mm shift bank B	1.079	1.077
10	Segments missing	0.997	0.993

First, in‐phantom dose measurements at isocenter using the ion chamber were done for delivery of the reference plan (Dmeas.ref) and the error‐induced plans (Dmeas.err, i; i: 1, ….. 10). Following the methodology described by Ezzell et al.,^(10)^ the conversion of chamber reading to dose was done by first irradiating the phantom with parallel‐opposed 10×10 fields arranged isocentrically and then establishing the ratio of reading to planned dose in that geometry. In this way, the effects of daily linac output variations and differences between the phantom and liquid water were reduced.

For each erroneous plan (plan.err1, plan.err10), a “reconstructed erroneous plan” (plan.

err1DYN, plan.err10DYN) was generated using the DynaLog files recorded during the irradiations. The chamber doses calculated by the TPS were registered for the reference plan (Dcalc.ref) and the erroneous plan (Dcalc.DYN, i, with i: 1,….,10).

The rational quantities Dcalc.DYN, i/Dcalc.ref and Dmeas.err, i/Dmeas.ref (with i: 1,.., 10) were compared (Table 1) and the Pearson correlation coefficient (R^2^) was calculated as an indicator of the accuracy of the DynaLog file‐based method in order to reproduce the actual dose delivery.

Secondly, the accuracy of the MATLAB code used to reconstruct the MLC pattern (dva generation) from the DynaLog files was evaluated using the methodology described in the Materials and Methods section C above. Moreover, we consider important to do a measurement‐based check of the accuracy of the fluences generated from the DynaLog files. For that, planar measurements were also made for all erroneous plans (plan.err1; …., plan.err10). These measurements were done separately from the chamber measurements. Each field was delivered to the EPID and the measured fluence was recorded. No phantom was present between the EPID and the beam. New reconstructed plans (plan.err1DYN2; .., plan.err10DYN2) were generated taking into account the DynaLog files recorded during the actual delivery.

The fluence at the EPID level was calculated (“predicted”) for each field of the reconstructed plans (plan.err1DYN2; …., plan.err10DYN2) by using the portal dose calculation (PDC) algorithm^(11)^ (v. 10.0, Varian). Accuracy of the PDC algorithm was assessed during its commissioning by using the Aida pattern.^(11)^ The gamma analysis^(12)^ revealed a passing rate of 99% for the criteria of 3% in relative dose and 3 mm as distance to agreement. Hence, the accuracy assigned to our portal dosimetry methodology was 3%/3 mm.

A predicted fluence of each field for each reconstructed plan (plan.err1DYN2; …., plan. err10DYN2) was computed by taking into account the MLC pattern reconstructed from the DynaLog files recorded during the same delivery as each fluence was measured. A quantitative assessment of the agreement between predicted and measured fluences was made using the gamma tool, using factors of 3% and 3 mm. The percentages of pixels with gamma less than 1.0 (gamma passing rate) were used to assess the accuracy of the DynaLog file‐based method to reproduce the actual fluence of a dynamic field as delivered by the linac.

### F. clinical application of dose reconstruction process

In order to illustrate the DynaLog file‐based reconstruction method, 20 clinical plans for four different sites (prostate, lung, breast, and head and neck) were reconstructed to compare for each case the dose distribution of the reference (original) plan to the recalculated one. The DVHs of the target volume were used to perform this comparison. The metrics minimum (D98%), maximum (D2%), and mean (Dmean) doses from the target DVH were registered for each original/reconstructed pair of plans. These doses were expressed as percentage of the prescription dose. The minimum and maximum doses were reported as D98% and D2%, respectively, according to the recommendations cited in ICRU Report 83.^(1)^

The dose differences in D2%, D98%, and Dmean may be used to quantify the “degree of agreement” between reconstructed and original plans that should be expected by using the “confidence limit”, as proposed by Palta et al.^(13)^ In their formulation, the confidence limit is the sum of the absolute value of the average difference and the standard deviation of the differences multiplied by a factor of 1.96. The confidence limit provides a mechanism for determining a reasonable action level for the patient DVH IMRT QA described in our work.

As additional information, the leaf positional deviations for all delivered fields of the 20 plans were analyzed by using the Varian application called DynaLog File Viewer (DFV) (version 6.5.1.6). DFV is a utility program that takes the data from the DynaLog files and converts the data into tables and plots of the leaf positional errors. A detailed description of the software can be found in the Varian reference guide.^(14)^

## III. RESULTS

### A. Accuracy of the DynaLog‐to‐dva conversion process

Negligible dose differences (within ±0.1%) for the target volumes and OARs were observed between the reference plan and the reconstructed plan for the three clinical plans analyzed (prostate, head and neck, and lung). This dose discrepancy indicates the inherent accuracy of the MATLAB‐based code used in this study. During the creation of a DynaLog file, multiple records are made for each segment because of the sampling process which is performed every 55 ms by the MLC controller. It was found that the interpolations of leaf positions and fractional doses are well handled by the MATLAB code used to generate MLC files (dva).

### B. Validation of dose reconstruction process

Table 1 shows the values for the ratios Dcalc.DYN/Dcalc.ref and Dmeas.err/Dmeas.ref that has been used in our study for evaluating the ability of the DynaLog‐based method to detect erroneous deliveries. There was a significant positive correlation $\left(R^{2}=0.9993;p<0.01\right)$ between both rational quantities, indicating that the DynaLog‐based reconstruction method is able to reproduce the variations in the dose due to errors occurring during the dynamic delivery by perturbing the planned beam fluence.

**Figure 2:**
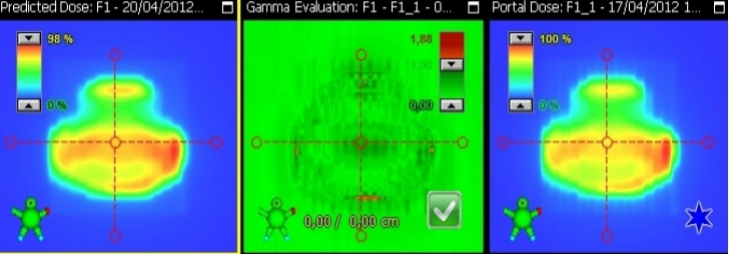
Predicted fluence (left) for a modulated field reconstructed according to the DynaLog files. The gamma analysis (middle) shows that the DynaLog‐based method is accurate enough to reproduce the delivered fluence (right).

The measured planar fluence at the level of the EPID was compared with the predicted one using a gamma passing criteria of 3%/3 mm for each field of each error‐induced plan (plan. err1,…, plan.err10). Predicted fluences were computed taking into account the recorded leaf positions given by the DynaLog files. The results of the gamma analysis are assessing the dva reconstruction method (as used in this work) in terms of fluence. An example of such a comparison is shown in Fig 2. The average value of the percentage of pixels passing the criteria was 99.9% (i.e., on the order of the passing rate registered during commissioning of the PDC algorithm).

### C. clinical application of dose reconstruction process

Table shows the maximum (D2%), minimum (D98%), and mean (Dmean) doses found in the target volume for each reference (original) plan and the reconstructed one, alongside the leaf positional error revealed by the DFV software after analysis of the DynaLog files recorded during delivery of the original plan. For each case, the maximum root mean square (RMS) error of each original plan is shown. The limit of confidence obtained from the dose difference data of Table 2 was 0.4 %, while the maximal error RMS value for leaf positional deviations ranged from 0.07 to 0.59 mm.

**Table 2:** Differences in parameter minimum dose (D98%), maximum dose (D2%), and mean dose (Dmean) for the target volume between the reference plan (original) and the DynaLog‐based reconstructed plan (recons.). Doses are expressed as percentage of the prescription dose. Analysis was performed for 20 clinical cases and four sites (head and neck, prostate, breast, and lung). Maximum root mean square error (RMS) is shown for each plan according DFV application

		*D98 % (%)*		*D2 % (%)*		*Dmean (%)*	
*Case*	*max RMS (cm)*	*Original*	*Recons.*	*Original*	*Recons.*	*Original*	*Recons.*
H&N#1	0.035	92.4	92.3	105.6	105.5	100.0	100.0
H&N#2	0.059	92.6	92.6	103.0	102.9	100.3	100.2
H&N#3	0.056	94.7	94.6	103.7	103.7	100.0	100.1
H&N#4	0.056	90.5	90.5	105.1	105.1	100.0	100.0
H&N#5	0.032	93.7	93.4	105.3	105.0	100.0	99.8
Pros#1	0.033	96.5	96.5	102.0	102.1	100.0	100.1
Prost#2	0.053	94.9	95.0	101.9	102.0	100.0	100.1
Prost#3	0.028	96.0	95.9	102.3	102.3	100.0	99.9
Prost#4	0.049	95.3	95.4	102.6	102.6	100.0	100.0
Prost#5	0.044	95.3	95.3	102.8	102.8	100.0	100.0
Breast#1	0.024	91.8	91.6	104.7	104.6	100.0	99.9
Breast#2	0.022	92.5	92.7	105.8	106.0	100.0	100.2
Breast#3	0.030	94.0	93.8	104.6	104.4	100.0	99.8
Breast#4	0.017	90.6	90.8	105.9	106.3	100.0	100.5
Breast#5	0.030	92.5	92.4	103.2	103.1	100.0	100.0
Lung#1	0.037	90.4	90.3	106.0	105.9	100.0	99.9
Lung#2	0.007	102.4	102.4	126.0	126.0	117.0	117.1
Lung#3	0.020	95.1	96.1	103.5	103.4	100.0	100.0
Lung#4	0.015	101.6	101.7	123.4	123.5	113.7	113.8
Lung#5	0.021	97.5	97.3	120.8	120.8	109.3	109.2

## IV. DISCUSSION

Since we started the IMRT technique in our department, the procedure for the patient‐related QA has been based on measurements as recommended by international guidelines. These have consisted of creating a hybrid plan with an acrylic phantom in order to do an experimental verification of the absolute dose at a selected point (i.e., as a way to check the monitor units given by the TPS). With a background of more than 1200 plan verifications, we have registered a mean deviation of 0.4% (SD: 2.0%) between the planned and measured point dose. The 95% confidence level of our point dose measurements is comparable to the data cited in the Report from AAPM Task Group 119 on IMRT commissioning.^(10)^ In addition, a check of the planned 2D fluences has been routinely done on a patient basis using portal dosimetry, with a gamma acceptance criteria of 3%/3 mm for 95% of the analyzed pixels. Our experience was always successful for clinical IMRT plans tested to date.

However, these two types of measurements are very time‐consuming when performed for each clinical IMRT plan. In addition, these measurement‐based checks have limitations in verifying the actual dose distribution received by the patient, because the patient's geometry is not used. On the other hand, recent publications^2^, ^15^ have alerted the community to the insensitivity of per‐field measurements (such as the portal measurement that we have done so far) to detect the clinical impact due to delivery errors. These facts have motivated us to move our patient‐related QA procedure to a DVH‐based one by using the DynaLog files provided by the linac. In 2004, Stell et al.^(16)^ presented a very interesting analysis of the DynaLog files for many clinical IMRT plans delivered at their institution with a commercial linac, in order to detect delivery errors related to the dynamic MLC. To illustrate the utility of using DynaLog files, they performed a DynaLog file‐based dose reconstruction for three patient cases where large delivery errors were detected. The recalculated plans were compared to the original plans in order to assess the potential clinical impact of these errors. The authors pointed out that there is no reason that a clinically significant error could not occur in a case with moderate delivery errors.

For the 20 clinical cases analyzed (Table 2), it was found that the differences in the parameters D98%, D2%, and Dmean for the target volume were always within 1.0%, when the reference (original) plan and the reconstructed plan were compared. Evidently, these results were obtained when an almost perfect dynamic beam delivery was done, as was also pointed out by the error RMS values for leaf positional deviations (ranged from 0.07 to 0.59 mm).

The limit of confidence obtained from the dose difference data of Table 2 was 0.4 %. Although the 1.96 multiplier used in the confidence limit calculation strictly applies when a very large number of samples is available, we have chosen an action level of 1.0% to be used; it as a significant difference in the DVH of the reconstructed plan with respect to the original one.

Therefore, we consider that, after comparing the DVHs for the target when the difference found in the parameters D98%, D2% or Dmean is larger than 1.0%, there is a reasonable doubt about the delivery of the clinical IMRT plan.

The recent work of Agnew et al.^(17)^ demonstrates the successful implementation of DynaLog files to validate IMRT deliveries and how DynaLog files can diagnose delivery errors not possible with phantom based QA. Their analysis was performed on planned and actual fluence maps reconstructed from the MLC control file and delivered DynaLog files, respectively. In contrast to our procedure, they did not perform a patient dose reconstruction in order to evaluate the clinical impact of the MLC delivery errors.

**Figure 3:**
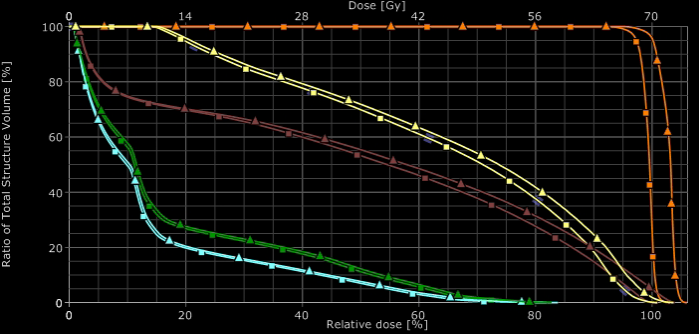
DVH‐based analysis between the reference plan (squares) and the DynaLog‐based reconstructed plan (triangles) for an intended error consisting of 0.5 mm MLC opening. Blue: right femoral head; green: left femoral head; brown: rectum; yellow: bladder; orange: target volume.

Figure 3 shows an example of evaluation of the clinical impact of a potential dosimetric error consisting of a 0.5 mm systematic expansion of the MLC opening. The differences in the doses for the target volume and the OARs between the reference plan and the reconstructed plan were significant. Therefore, the DynaLog‐based dose reconstruction could be a good procedure to analyze the clinical impact of possible MLC errors during a dynamic delivery.

Although the dva files generated to do the patient dose reconstruction were based on the recorded leaf positions and the recorded fractional MUs provided by the DynaLog files, they have some limitations. First, as DynaLog files only contain fractional MU information, they are not sensitive to errors related to a change on the absolute calibration of the linac. Zygmanski et al.^(18)^ have pointed out that DynaLog files do not reflect leaf positional errors related to a miscalibration (offset), as they rely on an internal measurement of the leaf positions. The authors have also stated that DynaLog files have limitations due to the inherent finite precision (±0.01 cm) and different sampling compared to sampling of the dva file planned for the delivered field. Luo et al.^(19)^ pointed out the potential inaccuracies of the DynaLog files if the encoders are miscalibrated. The MLC controller records the position of each leaf as MLC motor counts and a conversion factor is then used to convert motor counts to leaf positional information. Therefore, it is crucial to verify the MLC mechanical calibration in order to get reliable DynaLog files, so that the recorded leaf positions in the logs are the actual leaf positions. Also, it is highly recommended establishing a QA test to check the fractional MU data recorded in the DynaLog files, as this information is directly used to reconstruct the patient dose in our procedure. For this purpose, Li et al.^(5)^ have described an interesting methodology to perform this kind of verification. Therefore, we suggest the patient DVH‐based IMRT QA must be implemented alongside a specific QA program that checks the dynamic performance of the MLC, as well as the accuracy of the DynaLog file records.

The DynaLog‐based IMRT QA is currently performed in our department by using the DynaLog files recorded during the first treatment session of the patient, without a delivery in advance on a phantom. Analysis of the “delivered” dose distribution is always done before the second session in order to assure the correct delivery of the IMRT plan.

## V. CONCLUSIONS

We have shown a DynaLog file‐based method to perform patient plan‐specific IMRT QA taking into account the actual dynamic delivery and its influence on the patient dose distribution. According to our result, this method is reliable in order to detect potential errors during a dynamic delivery. The main issue of this method is its ability to translate the MLC delivery errors in terms of clinical impact. The method must be complemented with a dedicated MLC QA program that assures the accuracy of the MLC calibration.

Patient‐specific measurement procedures (e.g., hybrid plan, portal measurements) are currently reserved in our patient IMRT QA policy for the situation where the DynaLog‐based check reveals a considerable dose error.

## ACKNOWLEDGMENTS

The authors would like to thank Dr Jonas Bengtsson Scherman for the assistance he provided in designing the intended errors in the original MLC patterns used in this study.
